# Activating C–C Coupling on Copper during CO_2_RR: Charge-Controlled Design of Alloy Catalysts

**DOI:** 10.1021/acselectrochem.5c00297

**Published:** 2025-10-07

**Authors:** Wei Wang, Mattia Salomone, Michele Re Fiorentin, Francesca Risplendi, Giancarlo Cicero

**Affiliations:** Department of Applied Science and Technology, 19032 Politecnico di Torino, 10129 Turin, Italy

**Keywords:** electrochemical CO_2_ reduction, C−C coupling, copper-based alloys, density functional theory, constant potential, scaling relations

## Abstract

CO dimerization is a key step in the electrochemical reduction of CO_2_ to multicarbon (C_2+_) products at low overpotentials. Although Cu(100) is uniquely active for this process, its performance remains limited, and the mechanisms behind improved activity and selectivity through alloying are not fully understood. Here, we combine machine-learning screening with constant-potential density functional theory simulations to systematically investigate CO dimerization on dilute CuM(100) alloys. p-block metals, particularly Al and Ga, make the reaction exothermic and lower the activation barrier relative to pure Cu, with Al showing the highest activity. Charge analysis along the reaction path reveals that electron donation from these heteroatoms stabilizes the CO dimer intermediate, enabling efficient C–C coupling under operating conditions. This behavior is captured by a strong linear correlation between reaction energy and excess surface charge at fixed potential, introducing a physically grounded descriptor that integrates covalent and electrostatic contributions to reaction energetics. Our findings reveal that excess surface charge can serve as a practical reactivity descriptor that directly correlates with C–C coupling activity and guides the rational design of more efficient CO_2_RR electrocatalysts.

## Graphical abstract

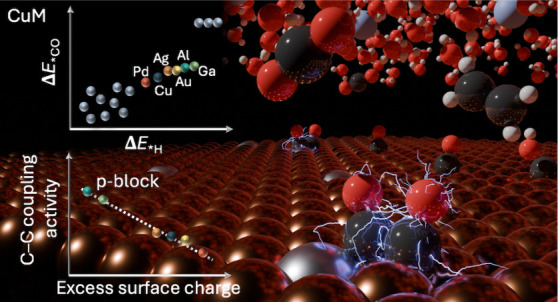

## Introduction

The electrochemical CO_2_ reduction reaction (CO_2_RR) is widely studied as a promising strategy for carbon-neutral chemical production, using renewable energy as a driving force to convert CO_2_ into value-added products, including single-carbon (C_1_) and multicarbon (C_2+_) species.^1–6^ However, electrocatalysts with high activity and selectivity are required for industrial-scale applications. Copper has been intensively studied for its unique property of catalyzing the formation of C_2+_ hydrocarbons and oxygenates at low overpotentials, especially on its (100) surface.^7–11^ This stems from the favorable binding energies of key intermediates, i.e., *CO and *H, which have been proposed as descriptors to assess the reactivity of a given catalytic surface.^12,65,66^ According to the Sabatier principle,^13^ the CO adsorption should be neither too weak, otherwise the CO desorbs before further reduction, nor too strong, as this could hinder subsequent steps and lead to catalyst poisoning. At the same time, the binding energy of hydrogen must also be considered: strong *H adsorption promotes the competing hydrogen evolution reaction (HER), reducing the overall selectivity toward CO_2_RR. Therefore, an ideal catalyst for C_2+_ production should exhibit moderate CO binding energy and weak *H binding energy, making these two descriptors essential criteria for identifying promising electrocatalyst candidates. Furthermore, the particular selectivity of the Cu(100) facet to reduce CO_2_ to C_2+_ products at low overpotentials has been attributed to its highly favorable CO dimerization, widely accepted as the rate-determining step, where the CO dimer preferentially forms at the hollow site via coupling of two CO molecules adsorbed on opposite bridge sites.^11,14,15^ Alternative pathways to C_2+_ products involving *CHO or *COH intermediates are generally recognized to become significant only at higher overpotentials.^16,17^

In this context, Cu-based electrocatalysts have been widely investigated to further enhance their performance towards C_2+_.^18,19^ Alloying Cu with other metals can modulate the surface electronic structure, intermediate adsorption energies (e.g., CO and H), and active site geometry, thereby tuning both activity and selectivity.^20,21^ Several studies^22,23^ have linked the improved C_2+_ selectivity of Cu-based alloys to more favorable energetics for key reaction steps, particularly C–C coupling. For example, Weitzner et al.^24^ reported that CuAl exhibits higher C_2+_ selectivity than pure Cu, which they attributed to enhanced thermodynamics of dimerization. Their analysis also showed that alloying elements such as B, Al, Ga, Pd, Ag, In, Sn, Pt, and Au tend to remain surface-exposed under electrochemical conditions due to favorable surface segregation energies. Liu et al.^25^ further examined the stability of copper surfaces with single impurity atoms (M) by computing binding energies and dissolution potentials of the M species. They found that most p-block and d-block elements (except Zn) have a strong affinity for the Cu surface, and identified CuAl and CuSi systems as particularly promising, combining stability with favorable adsorption energetics along the C_2_H_4_ formation pathway.

While compositional tuning offers a valuable route to optimize catalytic activity, a detailed understanding of these effects increasingly relies on first-principles simulations. Ab initio methods provide atomic-level insight into how alloying influences the energetics and mechanisms of CO_2_ reduction, especially the formation of multicarbon products. To generate accurate and transferable predictions, such simulations must also account for the influence of the electrochemical environment. Solvent effects and interfacial electric fields can significantly affect the thermodynamics and kinetics of key CO_2_RR steps, including CO dimerization.^26–29^ To address this, various modeling strategies have been developed, including solvation energy corrections,^8,30^ implicit solvent treatments,^16,17^ and explicit solvation models.^31–35^ For example, Santatiwongchai et al.^36^ showed that the interactions with water molecules greatly stabilize C–C coupling intermediates, yielding results that differ from those obtained in vacuum calculations. Similarly, Bagger et al.^35^ showed that the stabilization effect of the explicit aqueous phase makes the CO dimer stable only on the (100) facet, while it is not observed on the (111) and (110) facets. Electrode potential further modulates catalyst performance, particularly for multicarbon product formation. Experimental studies^37–39^ have shown that C_2+_ selectivity is highly potential-dependent, with Schouten et al.^9^ reporting a maximum in C_2_H_4_ production at −0.6 V vs the reversible hydrogen electrode (RHE) on Cu(100) at pH 7, ca. −1.1 V vs the standard hydrogen electrode (SHE). Together, these findings underscore the need for computational frameworks that explicitly incorporate solvation and applied potential, elements that are central to the approach adopted in this work.

In this paper, to rationally explore how catalyst composition influences CO dimerization activity, we focus on dilute CuM(100) alloys, where a substitutional metal heteroatom is introduced into the Cu(100) surface, which have been shown to be stable under electrochemical conditions.^24^ Our goal is to enhance the selectivity toward C_2+_ products by identifying the compositional features that facilitate CO dimerization at the experimentally relevant potential of −1.1 V vs SHE, where CO–CO coupling is the rate-determining step.^11,40,41^

Motivated by the central role of CO and H adsorption energies in determining catalytic performance,^12^ we employ a data-driven screening strategy to efficiently map reactivity trends across several CuM(100). By extending the machine-learning (ML) framework developed by Salomone et al.,^42^ we generate accurate predictions of *CO and *H binding energies for a broad set of CuM(100) surfaces, allowing us to rapidly identify promising alloy candidates. To complement this descriptor-based screening and evaluate catalytic performance under electrochemical conditions, we perform constant-potential density functional theory (DFT) calculations of the CO dimerization step. These simulations include solvation effects via an explicit water layer at the interface, providing a realistic description of the electrochemical environment. This combined ML–DFT approach allows us to evaluate both thermodynamic and kinetic factors governing C–C coupling at the electrochemical interface.

Our results provide the first systematic investigation of CO dimerization energetics and kinetics across a series of dilute CuM(100) alloy surfaces. We identify p-block elements, particularly Al and Ga, as highly promising heteroatoms that lower the dimerization barrier by stabilizing key intermediates through electron donation. Importantly, we find that the reaction energetics correlate strongly with the excess surface charge required to maintain the applied potential, introducing this quantity as a unifying descriptor that captures both covalent and electrostatic contributions. These insights demonstrate that catalyst evaluation must go beyond adsorption energies alone to include the electronic response of the surface under reaction conditions. Our results thus provide a mechanistic foundation and practical design principles for the development of more selective and efficient Cu-based electrocatalysts for CO_2_ reduction to multicarbon products.

## Methods

We employed a three-stage computational approach, combining adsorption energy calculations, machine learning predictions, and constant-potential DFT, to screen and analyze Cu-based alloys for their ability to promote CO dimerization. Starting from DFT-calculated *CO and *H adsorption energies on a set of dilute CuM alloy surfaces, we trained two-step ML models:^42^ a Gradient Boosting^43,44^ Classifier to identify stable adsorption sites, followed by a Gradient Boosting Regressor to predict adsorption energies across a broad alloy space. This enabled rapid identification of candidate surfaces with favorable adsorption characteristics for C_2+_ product formation: moderate *CO and weak *H binding. Selected alloys were then subject to detailed reaction pathway analysis using constant-potential DFT simulations,^45–47^ incorporating explicit solvation to capture key electrochemical effects relevant to CO dimerization.

All DFT calculations were performed using ultrasoft pseudopotentials^48^ and the Perdew–Burke–Ernzerhof (PBE)^49^ exchange-correlation functional with Monkhorst–Pack^50^ sampling within the Quantum Espresso^51,52^ code. The adsorption energies of *CO on 6 × 6 four-layered Cu_0.972_M_0.028_ surface slabs were adapted from Salomone et al.,^42^ while *H adsorption energies were calculated similarly.

To evaluate the CO dimerization step on selected candidate alloys, we performed constant-potential DFT calculations on 4 × 4 CuM(100) slabs (four layers) at −1.1 V vs SHE, consistently with the experimental applied potential favoring ethylene production, as reported in the introduction.^9^ To account for solvation, we employed a hybrid model with 10 explicit water molecules combined with an implicit continuum description provided by the ENVIRON^52,53^ plugin. This setup was chosen as the minimal explicit solvation necessary to capture local hydrogen-bonding and stabilization of adsorbates, while ensuring computational feasibility for a systematic alloy screening. Although this simplified treatment does not explicitly describe the Helmholtz layer, similar models have shown reasonable accuracy in reproducing interfacial effects in electrochemical simulations.^31,54–56^ We note that our conclusions rely on relative trends across alloy surfaces, which are expected to remain valid within this framework. Constant-potential DFT calculations were carried out using Quantum Espresso in combination with the Atomic Simulation Environment (ASE).^57^ Transition states (TSs) were identified at constant applied potential via the dimer method,^58^ with the initial guess obtained through the nudged elastic band (NEB) method^59,60^ at constant charge. The use of NEB and dimer methods with explicit solvating water molecules at constant potential provides a static identification of the TS and does not capture the full extent of solvent fluctuations. Nevertheless, previous studies have shown that constant-potential barrier calculations yield results consistent with constant-charge extrapolation approaches and experimental data.^36,47^ In our simulations, the water layer remains stable across the initial, transition, and final states (see the Supplementary Information), suggesting that solvent fluctuations do not introduce significant uncertainties. Since all alloy surfaces were treated within the same solvation framework, the comparative trends reported here can be regarded as robust.

Charge transfer was analyzed with both the Bader^61^ and the Löwdin^62^ partitioning schemes, using the projector-augmented wave (PAW) method^63^ with high wavefunction and density cutoffs, to better capture the contribution of inner electrons.

Further details on the DFT computational setup, ML implementation, adsorption energy, electronic grand-canonical energy and work function calculations are provided in the Supporting Information.

## Results and Discussion

### 1 Screening of Cu-Based Alloys via Machine Learning

The ML-predicted adsorption energies of the most stable *CO and *H configurations involving the impurity atom M on the CuM(100) surfaces are shown in Figure 1. For systems in which adsorption at the impurity is not stable and the adsorbate relaxes onto a neighboring Cu site during optimization, we label the configuration as “unstable”. These cases indicate that the impurity does not support stable adsorption. To illustrate this behavior, the Supporting Information, Figure S3, includes adsorption energy maps for *CO on two representative alloy surfaces: CuAl(100), where adsorption on the impurity is stable, and CuSn(100), where it is unstable and the adsorbate preferentially binds to adjacent Cu sites. These predictions reveal clear trends linked to the electronic nature of the substitutional atoms. Alloys containing post-transition or noble metals, such as Ag, Zn, Au, Al, Ga, and In, exhibit *CO and *H binding energies that are slightly weaker than those of pristine Cu. These characteristics are favorable for promoting C_2+_ product formation, as they reduce *CO poisoning and suppress the competing HER. In contrast, early and mid-transition metal impurities tend to strengthen *H adsorption, potentially shifting selectivity toward H_2_ and limiting CO surface coverage.

**Figure 1:**
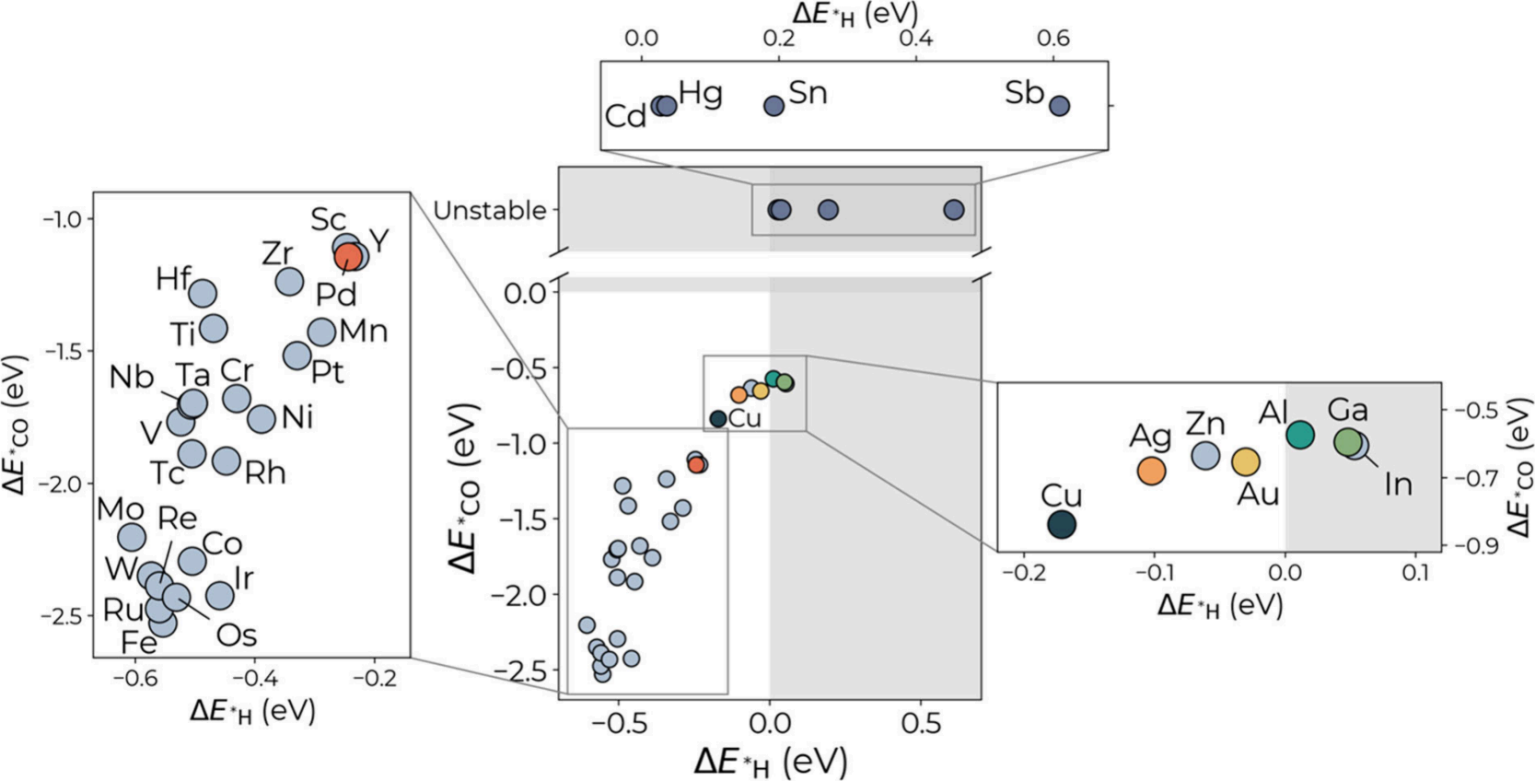
Lowest adsorption energies of CO (ΔE_*__CO_) and H (ΔE_*__H_) on pristine Cu(100) and CuM bimetallic (100) surfaces predicted by ML. The labels mark the species of the M heteroatom in the Cu(100) surface. All adsorption configurations on CuM(100) involve the impurity atom M. When adsorption on M is not stable and the adsorbate relaxes onto neighboring Cu sites, the configuration is labeled as “unstable”.

A few systems, including Cd, Hg, Sn, and Sb, show *CO instability and *H adsorption energies that are positive, indicating low likelihood of either CO reduction or HER, making them poor candidates for further investigation. For most systems, the impurity’s effect is localized: *H adsorption energies return to Cu-like values at sites farther than the first-nearest neighbor, similar to the behavior observed for *CO adsorption. In both (100) and (111) facets, hollow sites remain the most stable binding configuration for *H, consistent with previous studies.^64^

Based on the predicted *CO and *H adsorption energies, a group of Cu-based alloys, namely CuPd, CuAg, CuAu, CuAl, and CuGa, falls within the desired window of moderate *CO binding and weak *H binding identified in the literature^12,65,66^ and is therefore expected to enhance the C_2+_ product turnover. These systems are then selected for a detailed investigation of the CO dimerization energetics and kinetics under electrochemical conditions using constant-potential DFT. Their composition spans both transition and post-transition elements, enabling a comparative analysis of how electronic structure influences reactivity.

### 2 Activity of Dilute CuM(100) for CO Dimerization at Constant Potential

To gain mechanistic insight into CO dimerization and to verify the predictions from ML-based screening, we performed constant-potential DFT calculations on selected CuM(100) alloy surfaces, incorporating an explicit water layer to simulate electrochemical conditions. Pristine Cu was included as a reference for comparison. As suggested in Koper et al.’s work,^11,14,15^ CO dimerization preferentially occurs at sites with square symmetry. We consider as initial state for the reaction two CO molecules adsorbed at two opposite bridge sites between the copper and substitutional atom, with the CO dimer forming at the hollow site in between, as shown in panels a–c of Figure 2.

**Figure 2:**
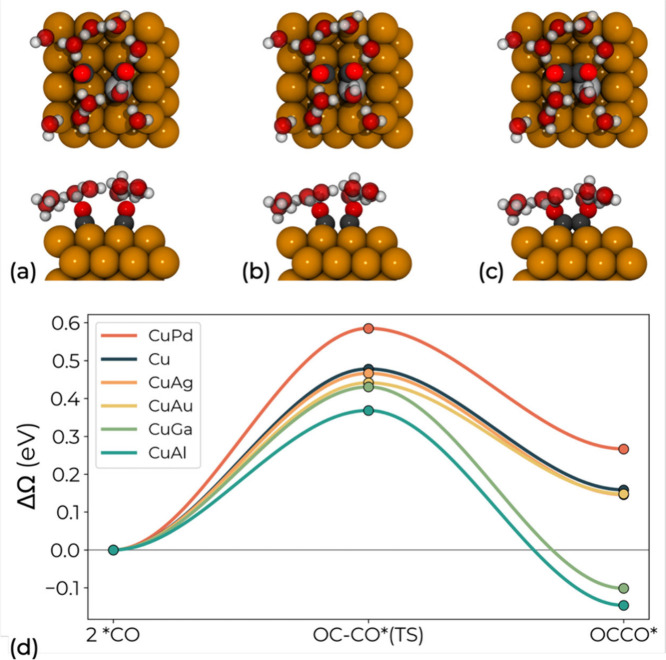
Top (upper panels) and side (lower panels) view of the initial (a), transition (b), and final (c) states of CO dimerization on a dilute CuM(100) alloy surface in the presence of a water layer. Two water molecules are omitted in the side view for clarity. H, C, O, Cu, and M substituent atoms are represented by white, dark grey, red, brown, and light grey, respectively. (d) Reaction energy profiles (in eV) for CO dimerization on Cu, CuAl, CuAg, CuAu, CuGa, and CuPd computed at −1.1 V vs SHE.

Figure 2d shows the energy profiles from initial, through transition to final state for CO dimerization on the CuM(100) surfaces at −1.1 V vs SHE. While the absolute energetics of CO–CO coupling varies with potential, becoming more favorable at more negative bias and less favorable at less negative bias, the relative trends across alloys are expected to remain robust, since they are governed by local charge redistribution and by the global electrostatic response of the surface. Relevant structural parameters, reaction energetics, computed interface capacitances and potentials of zero charge (PZCs) of the studied systems are respectively reported in Tables S3, S4, and S5 in the Supporting Information. Relative to pristine Cu, CuAg, CuAu, CuAl and CuGa, with weaker ΔE_*CO_, have lower activation barriers and reaction energies. Specifically, CuAl and CuGa have favorable energetics for CO dimerization, making the process exothermic (−0.15 and −0.10 eV, respectively), in line with experimentally observed C_2+_ performance improvements.^22,24,67^ CuAl is expected to show the best performances, with the lowest barrier (0.37 eV), 0.06 eV lower than that of CuGa. CuAg and CuAu have similar activation barriers (0.47 and 0.44 eV, respectively) and reaction energies (0.15 eV) compared to pure Cu whose activation and reaction energies are 0.48 and 0.16 eV, respectively. From their kinetics, diluted CuAg and CuAu alloys are expected to exhibit similar activity for CO dimerization as pure Cu. For CuPd, with stronger ΔE_*CO_, the initial state is the most stable, resulting in a higher barrier of 0.58 eV and reaction energy of 0.27 eV, showing less activity for C_2+_ formation.

To interpret the obtained energetics beyond adsorption behavior on CuM slabs, we calculated their work function, an electrostatic feature, and correlated it with the reaction energy of CO dimerization. As shown in Figure 3a, we find a correlation with an *R* ^2^ value of 0.78, where deviations from the linear trend align with the CO binding energy strength: Pd, with its stronger ΔE_*CO_, deviates positively, while Au, with its weaker ΔE_*CO_, deviates negatively. This indicates that the interplay between both electrostatic (work function) and covalent (CO binding energy) factors drives the reaction energetics, consistent with studies by Ringe^68^ and Chen et al.^69^ To capture contributions from both factors^70^ as well as capacitance and solvation, we evaluated the excess electrons required to bring the bare CuM(100) surface to a potential of −1.1 V_SHE_ under the same solvation environment. As shown in Figure 3b, CuM(100) alloys with p-block heteroatoms as Al and Ga draw approximately 0.90e from the external circuit, less than the ∼1e needed by alloys containing transition metals. This difference shows that, unlike transition metals, Al and Ga atoms in the Cu(100) surface readily donate electrons to the slab, reducing the need for externally supplied electrons to reach negative potentials. Across all studied CuM(100) alloys, the excess electrons on the slab correlates strongly with the reaction energy of CO dimerization, with a high *R* ^2^ = 0.99, demonstrating dominant electrostatic control of C–C coupling.

**Figure 3:**
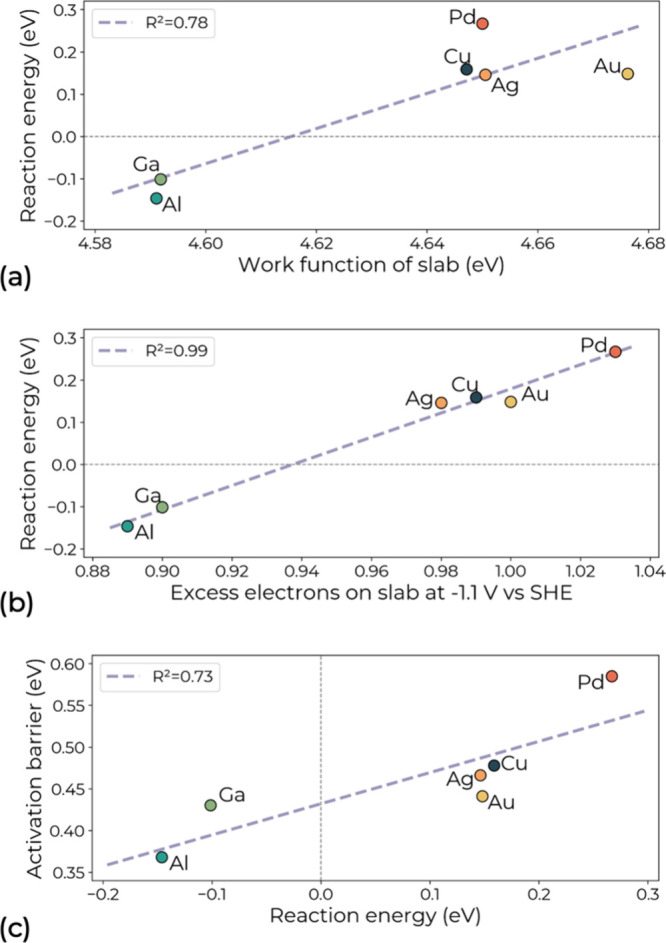
Scaling relations between (a) the reaction energy of CO dimerization and the calculated work function of Cu and CuM(100) slabs, (b) the reaction energy of CO dimerization and excess electrons of the slab at −1.1 V vs SHE, and (c) the activation barrier and the reaction energy for CO dimerization.

Figure 3c shows the correlation (*R* ^2^ = 0.73) between reaction and activation energies, confirming linear scaling consistent with a Brønsted–Evans–Polanyi relation.^71,72^ Combining this relation with the strong correlation between excess charge and reaction energy observed in Figure 3b, we identify the excess surface charge at the given applied potential as an effective descriptor for CO dimerization electrocatalyst screening. It shows a better correlation than either binding energy or work function in a vacuum, by incorporating contributions from both. Notably, a recent experimental study also reported that the surface charge of organic-functionalized Cu highly correlates with the yield of multicarbon products.^73^

Since, as shown, the reaction energetics of CO dimerization is strongly governed by electrostatics, we analyze the change along the reaction path of the Bader charge on the impurity atom M and on the adsorbed *CO molecules directly involved in the reaction, as marked in Figure 4a. Figure 4b shows the Bader charge on the M atom in the slab without adsorbates, both at the PZC and at −1.1 V_SHE_, and along the CO dimerization reaction. On the bare surfaces, the charge on the atom M becomes more negative with applied potential, while the neighboring Cu atom (Cu 1st) remains nearly neutral regardless of the applied potential (see Supporting Information, Figure S4). For transition metals Pd, Ag and Au, their higher electronegativity results in a greater accumulation of negative charge compared to neighboring Cu. In contrast, post-transition metals Al and Ga carry a more positive charge, as they easily donate electrons, consistent with our previous analysis. Most notably, Al exhibits the strongest electron donation, maintaining a Bader charge consistently above +1e. Charge transfer from the impurity atoms is further confirmed by the charge density differences reported in Figure S5. After the adsorption of two CO molecules, electrons are transferred from the surface to the 2*CO and the charge on M becomes less negative. The Cu atom becomes positively charged except in the case of CuAl. Correspondingly, the Bader charge on the adsorbed CO moieties, in Figure 4c,d, is almost the same for both fragments and consistently negative from the initial to the final reaction state. Pd, Ag, and Au behave like pure Cu, with the negative charge of the two *CO fragments becoming steadily more negative from reactants to products. This explains the similar reaction energetics for CuAg and CuAu to pure Cu. In contrast, the less favorable reaction energetics for CuPd can be attributed to its intrinsically strong CO binding energy. Al and Ga present an evidently different behavior with the *CO close to atom M, labeled CO(1), being more negatively charged than the other. Particularly, CO(1) in the CuAl alloy carries nearly twice the charge of the CO(2). Along the reaction path, more electrons transfer from the surface to the *CO molecules. Notably, from transition states to final states, the charge of 2*CO drops, suggesting that the exothermic reaction energies for CuAl and CuGa can be attributed to CO dimer stabilization through electron donation from metal atoms. Although Al donates more charge to the adjacent *CO than Ga, Figure 4c, both alloys display similar barriers and reaction energies (Figure 2) because their overall surface excess charge at −1.1 V vs SHE is comparable, as shown in Figure 3b. Al and Ga redistribute charge differently at the local scale but provide a similar net electrostatic stabilization, resulting in comparable reaction energetics.

**Figure 4:**
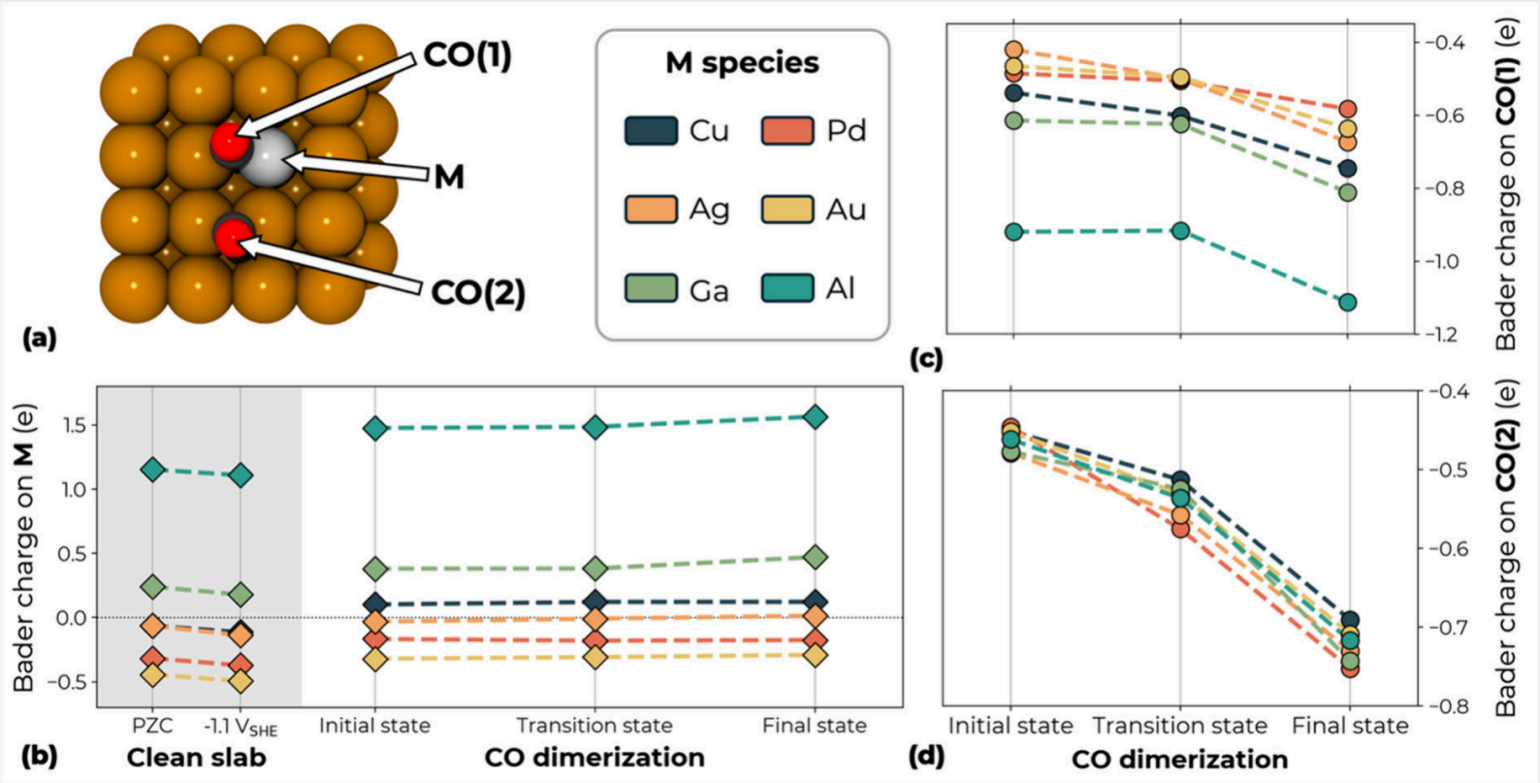
(a) Labeling of studied atoms for Bader charge analysis. (b) Bader charge of the impurity atom M in the clean slab without adsorbate, both at the PZC and at −1.1 V_SHE_, and along the CO dimerization path. (c) Bader charge along the dimerization path on the *CO molecule adsorbed on M, CO(1). (d) Bader charge along the dimerization path on the *CO molecule adsorbed on the neighboring Cu atom, CO(2).

The results obtained from the Löwdin charge analysis are reported for comparison in Figure S6 in the Supporting Information, and show that, while the absolute charge values obtained from the two schemes slightly differ, the overall trends are consistent. Moreover, correlations between local charge (Bader and Löwdin), excess surface charge and reaction energetics are shown in Figures S7 and S8 in the Supporting Information.

In general, reaction energetics for CO dimerization depend on both covalent (CO binding energy) and electrostatic (work function) descriptors, which are captured by the excess surface charge at a given potential. Transition metals Pd, Ag, and Au show similar charge transfer behavior to Cu along the reaction path, with the corresponding reaction energetics related to their CO binding energies, i.e. unfavorable for CuPd with stronger ΔE_*CO_. p-block metals Al and Ga donate more electrons to stabilize 2*CO molecule during the dimerization, making the process spontaneous.

## Conclusions

We investigated how dilute Cu-based alloys affect CO dimerization under electrochemical conditions, combining machine learning and constant-potential DFT simulations. Among the screened candidates, CuAl and CuGa stood out by making CO dimerization exothermic and with low kinetic barrier at −1.1 V vs SHE, while CuPd showed poor activity due to overly strong CO binding. CuAg and CuAu displayed similar behavior to pure Cu despite weaker *CO adsorption, highlighting that adsorption strength alone does not dictate reactivity. Crucially, we showed that post-transition metals like Al and Ga promote *CO dimerization via electron donation, confirmed through Bader charge analysis, revealing the importance of electronic response beyond traditional adsorption descriptors.

Our findings establish general design principles for advancing electrocatalysts toward selective C_2+_ product formation, extending beyond the specific case of CuM(100) alloys. We show that efficient C–C coupling requires not only favorable adsorption energies, but also a surface environment capable of stabilizing charged intermediates under operating conditions. Importantly, we identify the excess surface charge at constant potential as a reliable and transferable descriptor that captures both covalent and electrostatic contributions to reactivity. Although Cu-based surfaces are known to undergo dynamic reconstruction under CO_2_RR conditions,^74,75^ and similar effects are expected in Cu-based alloys, this study specifically focuses on the local environment around the alloying atom to understand its electronic influence on catalytic reactivity. Broader surface transformations that alter morphology and active site geometry fall outside our scope. Despite this simplification, our results demonstrate that local charge donation by p-block elements plays a decisive role in stabilizing CO intermediates and promoting C–C coupling, suggesting that the excess surface charge descriptor remains a meaningful and predictive parameter, even in the presence of surface dynamics. This descriptor can be readily computed and used as a pre-screening filter to prioritize catalyst candidates prior to more expensive kinetic modeling. To meet the dual requirements of intermediate stabilization and charge transfer, alloying with electropositive, electron-donating elements, such as p-block metals, emerges as an effective strategy. However, similar effects may be achieved through alternative surface modifications,^76^ including the introduction of undercoordinated sites, vacancies, or impurity-induced distortions that localize charge and stabilize reaction intermediates. Electrochemical tuning^77^ offers additional pathways: conductive supports, high-permittivity electrolytes, or materials with intrinsically high PZC can promote surface charging, potentially activating otherwise marginal catalyst surfaces. Together, these insights define a comprehensive framework for catalyst design that integrates composition, surface structure, and electrochemical environment engineering for the rational development of next-generation systems for CO_2_ reduction to multicarbon products.

## Supplementary Material



## Data Availability

Computational data underlying this study can be accessed via the ioChem-BD data repository^78^ at 10.19061/iochem-bd-6-546.

## Notes

Computational details, implementation and performance of the ML models, and DFT results (PDF)

## Abbreviations

ASE: atomic simulation environment
CO_2_RR: CO_2_ reduction reaction
C_1_: single-carbon
C_2+_: multicarbon
DFT: density functional theory
HER: hydrogen evolution reaction
ML: machine learning
PBE: Perdew–Burke–Ernzerhof
PZC: potential of zero charge
RHE: reversible hydrogen electrode
SHE: standard hydrogen electrode
TS: transition state
