# Supplementary material for: Activating C–C Coupling on Copper during CO2RR: Charge-Controlled Design of Alloy Catalysts
Source: ACS Electrochem. 2025 Oct 7;1(11):2512–20. doi: 10.1021/acselectrochem.5c00297 (PMC12598899; doi:10.1021/acselectrochem.5c00297)
Supplement: Supplementary file 1 [file ec5c00297_si_001.pdf]

Supporting Information: Activating C–C Coupling on Copper during CO_2RR: Charge-Controlled Design of Alloy Catalysts

Wei Wang,* Mattia Salomone, Michele Re Fiorentin, Francesca Risplendi, and Giancarlo Cicero

Department of Applied Science and Technology, Politecnico di Torino, 10129 Turin, Italy

E-mail: wei.wang@polito.it

Contents

Computational details **S-1**

Implementation and performance of the ML models **S-3**

DFT results **S-6**

Computational details

All density functional theory (DFT) calculations were performed using ultrasoft pseudopotentials[S1] and the Perdew-Burke-Ernzerhof (PBE)[S2] exchange correlation functional within the Quantum Espresso[S3,S4] code. The energy cutoff was set to 40 Ry for wavefunctions and

400 Ry for the charge density and potential. For charge analysis, the projector augmented wave (PAW) method[S5] was employed to describe the effect of the inner electrons, with an energy cutoff of 60 Ry for wavefunctions and 600 Ry for the charge density and potential.

The adsorption energies of *CO were adapted from Salomone et al.,[S6] calculated on 6×6 four-layered $Cu_{0.972}M_{0.028}$ slabs with a vacuum layer of 12 Å. The adsorption energies of *H were calculated in the same way, where the bottom two layers were fixed. A 2 × 2 × 1 Monkhorst–Pack[S7] k-point grid was employed for Brillouin zone sampling. The adsorption energy of intermediates X (X = CO, H) was calculated as

$$\Delta E_{*\mathrm{X}} = E_{\mathrm{slab}+*\mathrm{X}} - E_{\mathrm{slab}} - E_{\mathrm{X}} \tag{1}$$

where $E_{\mathrm{slab}+*\mathrm{X}}$ is the calculated energy of the slab with the adsorbed intermediate, E_{slab} is the energy of the clean slab, E_{X} represents the energy of the isolated CO molecule or half the energy of the H_2 molecule. The study included a series of substitutional impurities (M = Al, Ti, V, Fe, Co, Ni, Zn, Mo, Pd, Ag, Sn, Sb, Pt, Au), selected to span a range of electronic and chemical characteristics from transition and post-transition metals. A total of 1564 adsorption configurations were computed across various CuM(100)/(111) surfaces. The DFT adsorption energies were then used to train two-step ML models to predict *CO and *H adsorption energies on a broader set of CuM surfaces as described in the following section.

To investigate CO dimerization on selected candidate alloys, constant-potential DFT calculations were performed on 4×4 CuM(100) slabs (four layers, with the bottom two layers fixed) at –1.1 V vs SHE. The Brillouin zone was sampled using a 3 × 3 × 1 Monkhorst–Pack grid to ensure consistent k-point density. These calculations included an explicit water layer (10 water molecules) and implicit solvation using the ENVIRON[S4,S8] plugin, which implements solvent-aware solvation models. Transition states (TS) for CO dimerization were identified using the dimer method[S9] at constant applied potential, with the initial guess gen-

erated by the nudged elastic band method[S10,S11] at constant charge. The structures of reactant and product were relaxed until atomic forces were below 0.05 eV/Å. For the transition state search, the same force threshold was applied. These constant-potential DFT calculations were carried out using Quantum Espresso in combination with the Atomic Simulation Environment (ASE).[S12] The electronic grand canonical energy Ω of reaction intermediate with different number of electrons at constant potential U was calculated as[S13–S15]

$$\Omega = E_{\mathrm{DFT}} - N_e \mu_e(U) \tag{2}$$

where E_{DFT} is the electronic energy obtained by DFT calculations, N_e is the number of added/removed electrons, and electronic chemical potential μ_e is referenced to the SHE scale (4.44 V from experimental measurements[S16]) as

$$\mu_e = -\Phi_{\mathrm{SHE}} - U \tag{3}$$

Additionally, the work function Φ of the selected alloy surfaces was evaluated in vacuum as

$$\Phi = e\phi_{\mathrm{vacuum}} - E_{\mathrm{Fermi}} \tag{4}$$

where ϕ_{vacuum} is electrostatic potential in vacuum, e is the elementary charge, and E_{Fermi} is the Fermi level of the surface.

Implementation and performance of the ML models

To predict CO and H adsorption energies on CuM alloy surfaces, we adapted the two-step ML approach proposed in our recent work.[S6] We employed the Gradient Boosting[S17,S18] Classifier (GBC) and Regressor (GBR) for their superior performance. First, a Gradient Boosting Classifier was trained to identify stable adsorption sites. Subsequently, a Gradient Boosting Regressor was trained to estimate the adsorption energies at these stable sites. The

dataset was split into 80% for training and 20% for testing. To optimize the hyperparameters (listed in Table S1), we employed an 8-fold cross-validation.

The performance of the trained machine learning models was evaluated using the F1 score for classification and the root mean squared error (RMSE) along with the coefficient of determination (R^2) for regression, as summarized in Table S2. Higher F1 and R^2 values (closer to 1) indicate better model performance in classification and regression tasks, respectively. In addition, we report the receiver operating characteristic (ROC) curve obtained with the GBC model and the parity plot produced by the GBR model in Figures S1 and S2, respectively. The ROC curve plots the true positive rate (TPR) against the false positive rate (FPR) for different classification thresholds. These quantities are defined as:

$$\text{TPR} = \frac{TP}{TP + FN} \qquad \text{and} \qquad \text{FPR} = \frac{FP}{FP + TN}$$

where TP, FN, FP, and TN represent true positives, false negatives, false positives, and true negatives, respectively. The greater the distance from the diagonal line corresponding to a random classifier (dashed black line), the better the classification performance of the model. The ROC curve rapidly approaches the top-left corner, indicating a high TPR (close to 1) and a low FPR (close to 0) over a range of thresholds, which further confirms the strong predictive capability of the model. In the case of regression, the parity plot illustrates the predicted versus DFT-calculated CO and H adsorption energies on the test set. Points closely aligned with the $y = x$ diagonal indicate high model accuracy. Since all points lie near this line, the model demonstrates excellent predictive performance across the entire range of adsorption energies. These results confirm that both the classification and regression models perform effectively.

Table S1: Hyperparameters for the Gradient Boosting Classifier (GBC) and Gradient Boosting Regressor (GBR) models determined using 8-fold cross-validation.

Algorithm	Hyperparameters
GBC	max_depth = 8, min_samples_leaf = 3, n_estimators = 18
GBR	learning_rate = 0.18, max_depth = 5, max_features = 15, n_estimators = 29

Table S2: F1 scores of the Gradient Boosting Classifier (GBC) for the majority (Maj) and minority (Min) classes, as well as the root mean squared error (RMSE) and coefficient of determination (R^2) for the Gradient Boosting Regressor (GBR).

**Classification Algorithm**	**Training**		**Test**	
	**Maj**	**Min**	**Maj**	**Min**
GBC	0.99	0.98	0.97	0.88
**Regression Algorithm**	**Training**		**Test**	
	$\mathbf{R^2}$	**RMSE (eV)**	$\mathbf{R^2}$	**RMSE (eV)**
GBR	0.99	0.014	0.97	0.058

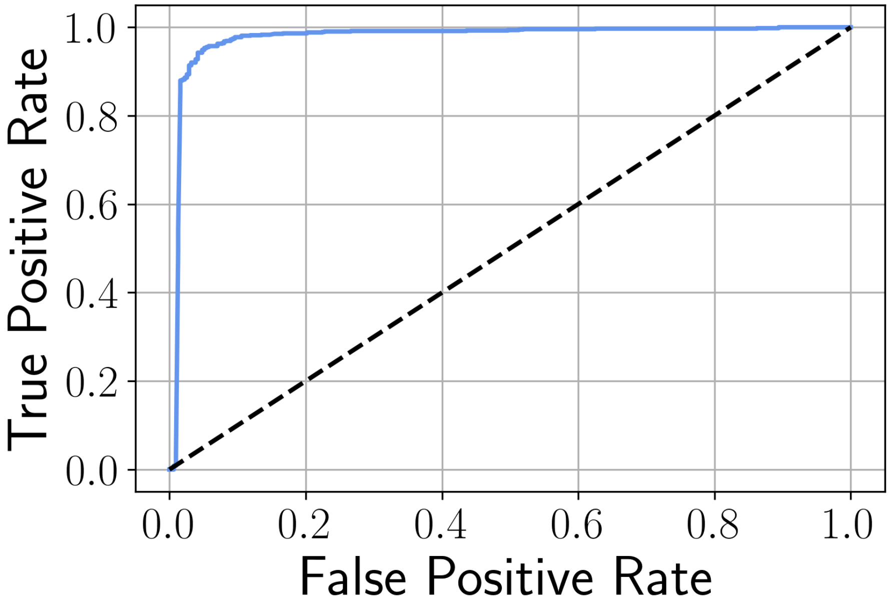

Figure S1: True Positive Rate vs False Positive Rate for the GBC. The greater the distance from the pure random classifier line (dashed black line), the better the model performance.

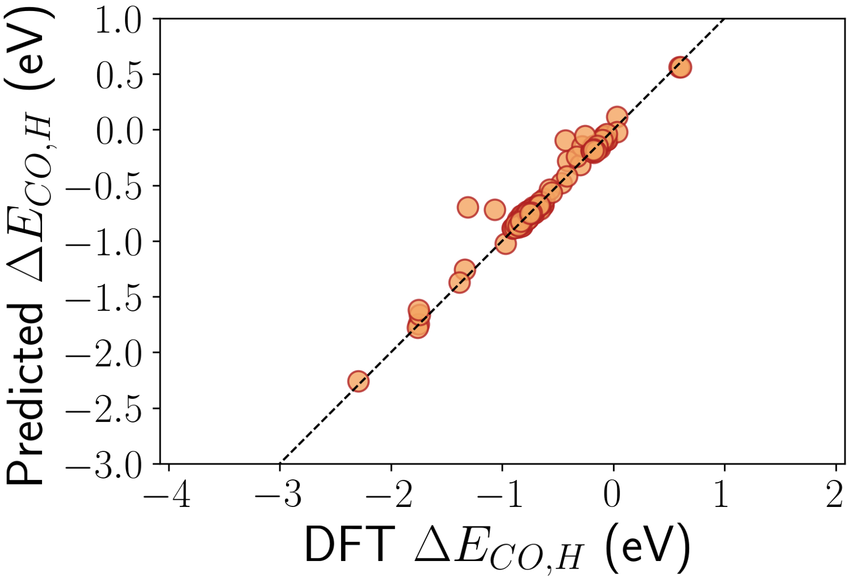

Figure S2: Relationship between $\Delta E_{CO,H}$ values predicted by the GBR and the DFT-calculated CO/H adsorption energies. The closer the points are to the dashed black line, the better the model performs.

DFT results

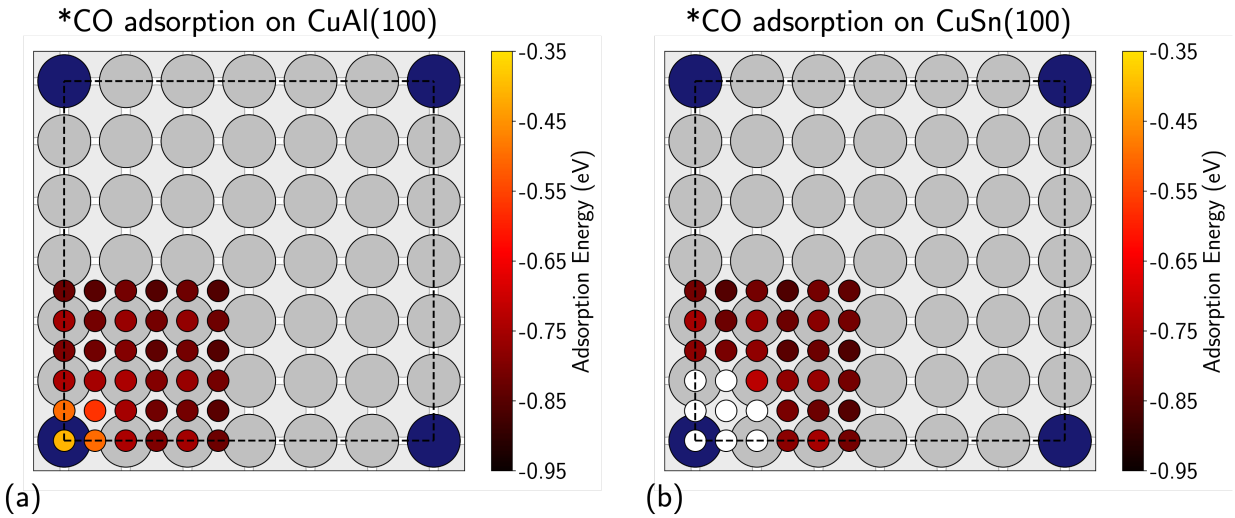

Figure S3: Map of *CO adsorption energy on different sites in CuM(100) dilute alloys. (a) Adsorption on CuAl(100). (b) Adsorption on CuSn(100). White circles mark unstable adsorption sites.

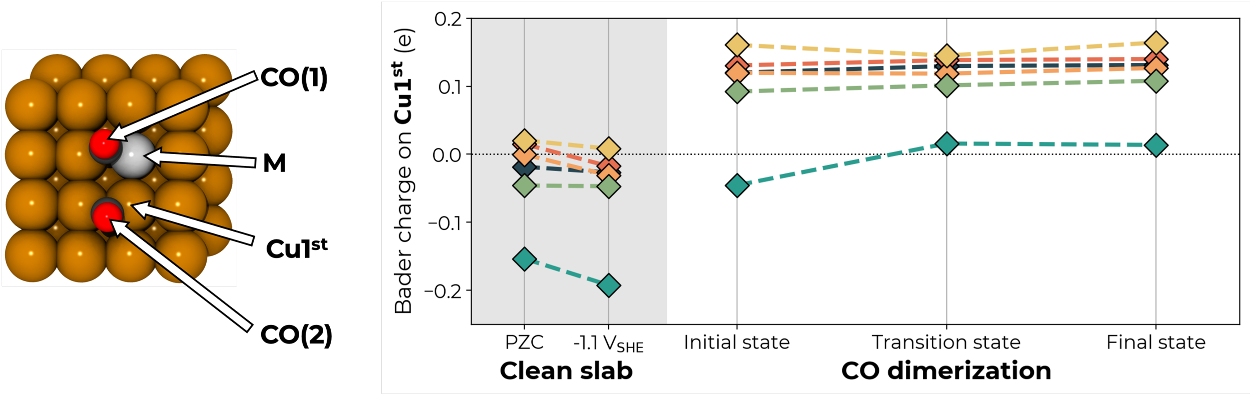

Figure S4: Bader charge on the Cu atom neighboring the impurity M ($Cu1^{st}$) on the clean slab without adsorbates, both at the potential of zero charge (PZC) and at –1.1 V vs. SHE, and along CO dimerization.

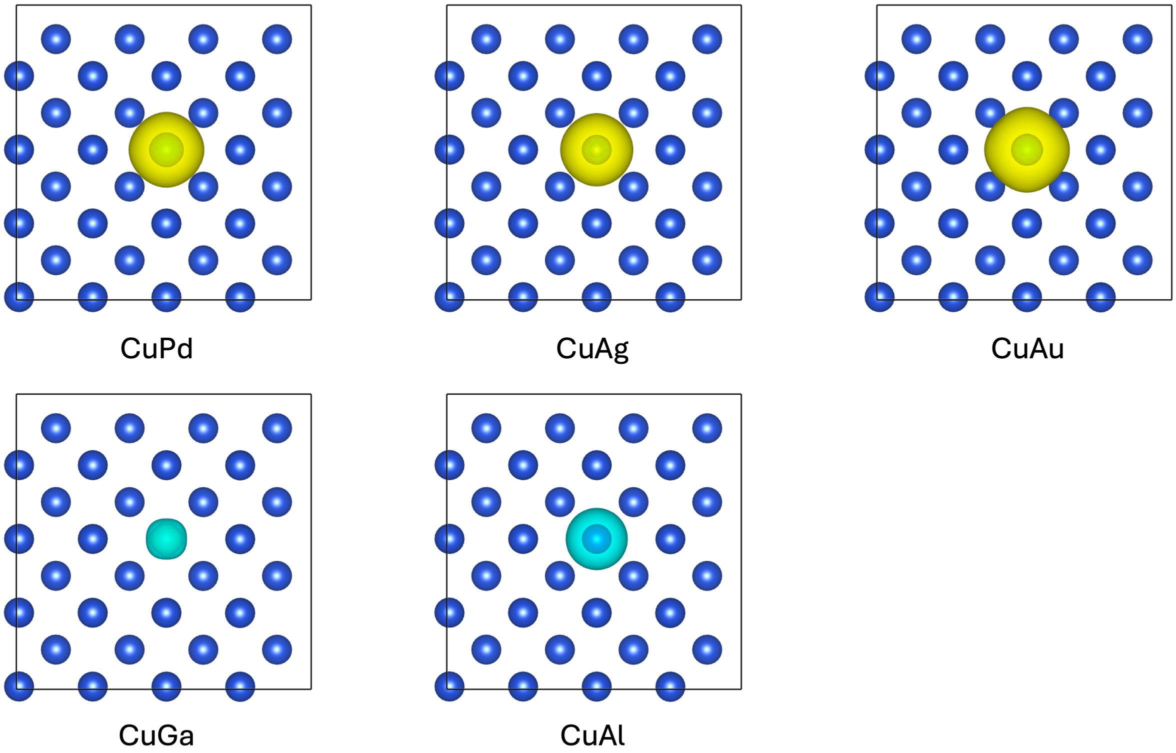

Figure S5: Charge density difference plots for CuM(100). Yellow and blue regions indicate charge accumulation and depletion, respectively. The isosurface value is 0.001 a.u.

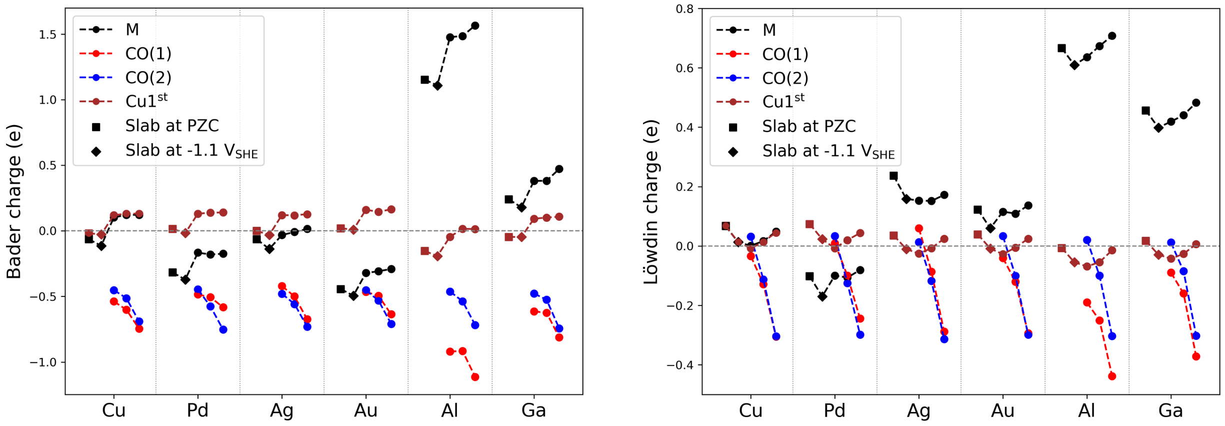

Figure S6: Bader and Löwdin charges of the clean slabs at the potential of zero charge (PZC, squares), at -1.1 V_{SHE} (diamonds), and along the CO dimerization pathway (circles, from initial to final state).

Table S3: Structural parameters of CO adsorbed on M (CO(1)) and on Cu (CO(2)) in the initial state (IS), transition state (TS), and final state (FS) along the dimerization pathway at -1.1 V_{SHE}.

CuM(100)		d_{C-C} (Å)	$d_{C-O(1)}$ (Å)	$d_{C-O(2)}$ (Å)
Cu	IS	3.35	1.21	1.19
	TS	2.03	1.22	1.22
	FS	1.48	1.29	1.29
Pd	IS	3.37	1.21	1.19
	TS	2.01	1.23	1.22
	FS	1.48	1.28	1.29
Ag	IS	3.39	1.19	1.19
	TS	2.05	1.21	1.22
	FS	1.49	1.28	1.29
Au	IS	3.36	1.20	1.19
	TS	2.05	1.22	1.22
	FS	1.48	1.28	1.29
Al	IS	3.38	1.22	1.19
	TS	2.10	1.23	1.22
	FS	1.47	1.30	1.29
Ga	IS	3.51	1.21	1.19
	TS	2.11	1.22	1.21
	FS	1.48	1.30	1.29

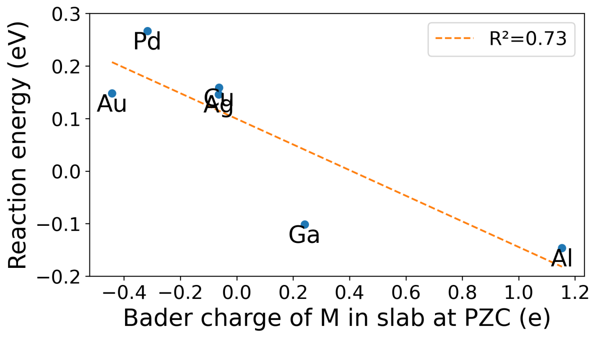

(a)

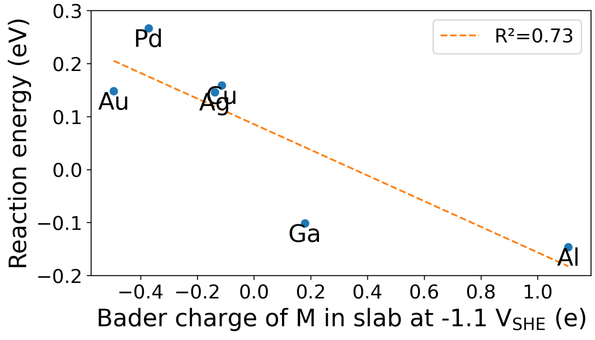

(b)

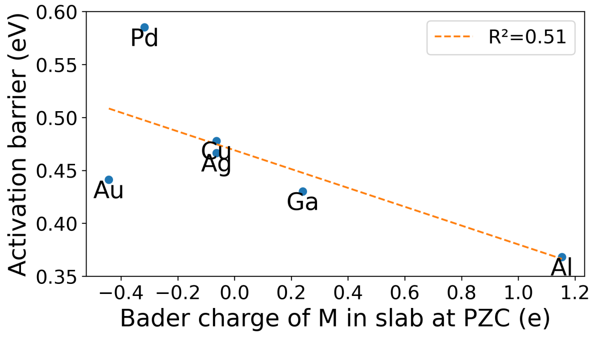

(c)

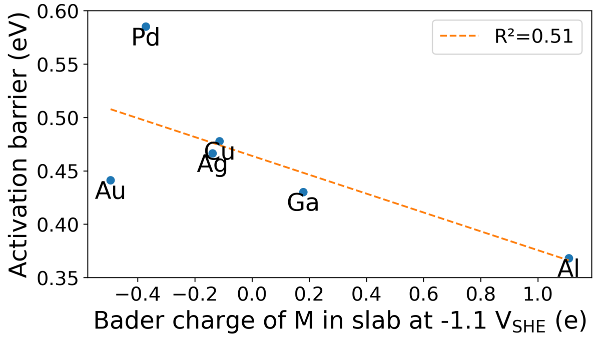

(d)

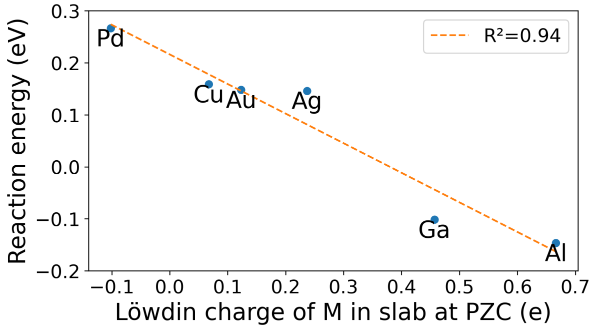

(e)

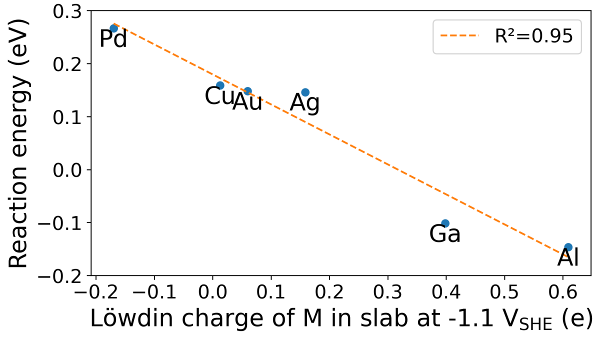

(f)

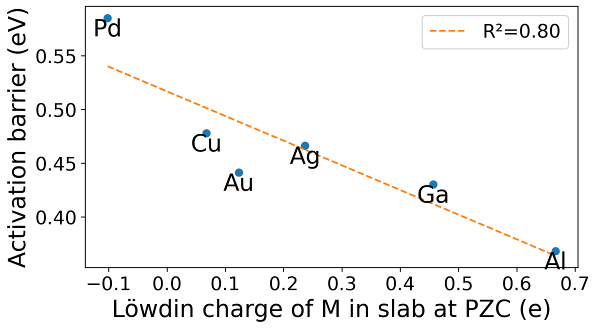

(g)

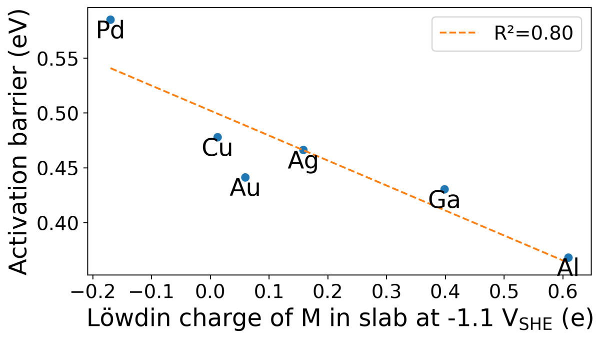

(h)

Figure S7: Scaling relations between CO dimerization energetics and the charges of the substitutional atom (M) in the slab: (a)–(b) reaction energy vs. Bader charge at PZC and –1.1 V_{SHE}; (c)–(d) activation barrier vs. Bader charge at PZC and –1.1 V_{SHE}; (e)–(f) reaction energy vs. Löwdin charge at PZC and –1.1 V_{SHE}; (g)–(h) activation barrier vs. Löwdin charge at PZC and –1.1 V_{SHE}.

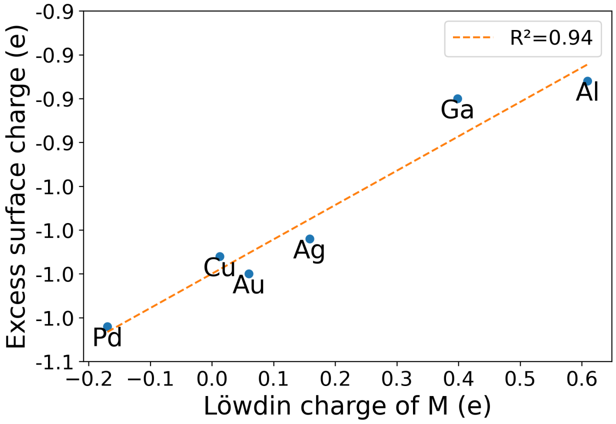

Figure S8: Correlation between excess surface charge and Löwdin charge of M in CuM(100) slabs at –1.1 V_{SHE}.

Table S4: The calculated reaction energy and activation barrier for CO dimerization on Cu and CuM(100) surfaces at –1.1 V_{SHE}.

CuM(100)	Reaction energy (eV)	Activation barrier (eV)
Cu	0.16	0.48
Pd	0.27	0.58
Ag	0.15	0.47
Au	0.15	0.44
Al	-0.15	0.37
Ga	-0.10	0.43

Table S5: The calculated capacitance and potential of zero charge (PZC) of Cu and CuM(100) surfaces.

CuM(100)	Capacitance (e/V)	PZC (V vs SHE)
Cu	1.6533	-0.0850
Pd	1.7432	-0.0801
Ag	1.6662	-0.0803
Au	1.6656	-0.0588
Al	1.6767	-0.1442
Ga	1.7015	-0.1417

References

(S1) Schlipf, M.; Gygi, F. Optimization algorithm for the generation of ONCV pseudopotentials. *Computer Physics Communications* **2015**, *196*, 36–44.

(S2) Perdew, J. P.; Burke, K.; Wang, Y. Generalized gradient approximation for the exchange-correlation hole of a many-electron system. *Physical Review B* **1996**, *54*, 16533–16539.

(S3) Giannozzi, P.; Baroni, S.; Bonini, N.; Calandra, M.; Car, R.; Cavazzoni, C.; Ceresoli, D.; Chiarotti, G. L.; Cococcioni, M.; Dabo, I.; Dal Corso, A.; de Gironcoli, S.; Fabris, S.; Fratesi, G.; Gebauer, R.; Gerstmann, U.; Gougoussis, C.; Kokalj, A.; Lazzeri, M.; Martin-Samos, L.; Marzari, N.; Mauri, F.; Mazzarello, R.; Paolini, S.; Pasquarello, A.; Paulatto, L.; Sbraccia, C.; Scandolo, S.; Sclauzero, G.; Seitsonen, A. P.; Smogunov, A.; Umari, P.; Wentzcovitch, R. M. QUANTUM ESPRESSO: a modular and open-source software project for quantum simulations of materials. *Journal of Physics: Condensed Matter* **2009**, *21*, 395502.

(S4) Giannozzi, P.; Andreussi, O.; Brumme, T.; Bunau, O.; Buongiorno Nardelli, M.; Calandra, M.; Car, R.; Cavazzoni, C.; Ceresoli, D.; Cococcioni, M.; Colonna, N.; Carnimeo, I.; Dal Corso, A.; de Gironcoli, S.; Delugas, P.; DiStasio, R. A.; Ferretti, A.; Floris, A.; Fratesi, G.; Fugallo, G.; Gebauer, R.; Gerstmann, U.; Giustino, F.; Gorni, T.; Jia, J.; Kawamura, M.; Ko, H.-Y.; Kokalj, A.; Küçükbenli, E.; Lazzeri, M.; Marsili, M.; Marzari, N.; Mauri, F.; Nguyen, N. L.; Nguyen, H.-V.; Otero-de-la Roza, A.; Paulatto, L.; Poncé, S.; Rocca, D.; Sabatini, R.; Santra, B.; Schlipf, M.; Seitsonen, A. P.; Smogunov, A.; Timrov, I.; Thonhauser, T.; Umari, P.; Vast, N.; Wu, X.; Baroni, S. Advanced capabilities for materials modelling with Quantum ESPRESSO. *Journal of Physics: Condensed Matter* **2017**, *29*, 465901.

(S5) Blöchl, P. E. Projector augmented-wave method. *Physical Review B* **1994**, *50*, 17953–17979.

(S6) Salomone, M.; Fiorentin, M. R.; Risplendi, F.; Raffone, F.; Sommer, T.; García-

Melchor, M.; Cicero, G. Efficient mapping of CO adsorption on $Cu_{1-x}M_x$ bimetallic alloys via machine learning. *Journal of Materials Chemistry A* **2024**, *12*, 14148–14158.

(S7) Monkhorst, H. J.; Pack, J. D. Special points for Brillouin-zone integrations. *Physical Review B* **1976**, *13*, 5188–5192.

(S8) Andreussi, O.; Dabo, I.; Marzari, N. Revised self-consistent continuum solvation in electronic-structure calculations. *The Journal of Chemical Physics* **2012**, *136*, 064102.

(S9) Henkelman, G.; Jónsson, H. A dimer method for finding saddle points on high dimensional potential surfaces using only first derivatives. *The Journal of Chemical Physics* **1999**, *111*, 7010–7022.

(S10) Mills, G.; Jónsson, H.; Schenter, G. K. Reversible work transition state theory: application to dissociative adsorption of hydrogen. *Surface Science* **1995**, *324*, 305–337.

(S11) Jónsson, H.; Mills, G.; Jacobsen, K. W. *Classical and Quantum Dynamics in Condensed Phase Simulations*; WORLD SCIENTIFIC, 1998; pp 385–404.

(S12) Hjorth Larsen, A.; Jørgen Mortensen, J.; Blomqvist, J.; Castelli, I. E.; Christensen, R.; Dułak, M.; Friis, J.; Groves, M. N.; Hammer, B.; Hargus, C.; Hermes, E. D.; Jennings, P. C.; Bjerre Jensen, P.; Kermode, J.; Kitchin, J. R.; Leonhard Kolsbjerg, E.; Kubal, J.; Kaasbjerg, K.; Lysgaard, S.; Bergmann Maronsson, J.; Maxson, T.; Olsen, T.; Pastewka, L.; Peterson, A.; Rostgaard, C.; Schiøtz, J.; Schütt, O.; Strange, M.; Thygesen, K. S.; Vegge, T.; Vilhelmsen, L.; Walter, M.; Zeng, Z.; Jacobsen, K. W. The atomic simulation environment—a Python library for working with atoms. *Journal of Physics: Condensed Matter* **2017**, *29*, 273002.

(S13) Hagopian, A.; Doublet, M.-L.; Filhol, J.-S.; Binninger, T. Advancement of the Homogeneous Background Method for the Computational Simulation of Electrochemical Interfaces. *Journal of Chemical Theory and Computation* **2022**, *18*, 1883–1893.

(S14) Hörmann, N. G.; Andreussi, O.; Marzari, N. Grand canonical simulations of electrochemical interfaces in implicit solvation models. *The Journal of Chemical Physics* **2019**, *150*, 041730.

(S15) Van den Bossche, M.; Skúlason, E.; Rose-Petruck, C.; Jónsson, H. Assessment of Constant-Potential Implicit Solvation Calculations of Electrochemical Energy Barriers for H_2 Evolution on Pt. *The Journal of Physical Chemistry C* **2019**, *123*, 4116–4124.

(S16) Trasatti, S. The absolute electrode potential: an explanatory note (Recommendations 1986). *Pure and Applied Chemistry* **1986**, *58*, 955–966.

(S17) Friedman, J. H. Greedy function approximation: A gradient boosting machine. *The Annals of Statistics* **2001**, *29*, 1189–1232.

(S18) Friedman, J. H. Stochastic gradient boosting. *Computational Statistics & Data Analysis* **2002**, *38*, 367–378.
